# Crystal structure of 2-methyl-*N*-{[2-(pyri­din-2-yl)eth­yl]carbamo­thio­yl}benzamide

**DOI:** 10.1107/S2056989015013559

**Published:** 2015-08-06

**Authors:** Nadiah Ameram, Farook Adam

**Affiliations:** a School of Chemical Sciences, Universiti Sains Malaysia, 11800 Georgetown, Penang, Malaysia

**Keywords:** crystal structure, benzamide, carbonyl thio­urea, inversion dimers, hydrogen bonding

## Abstract

In the title compound, C_16_H_17_N_3_OS, a benzoyl thio­urea derivative, the planes of the pyridine and benzene rings are inclined to one another by 66.54 (9)°. There is an intra­molecular N—H⋯O hydrogen bond present forming an *S*(6) ring motif. In the crystal, mol­ecules are linked *via* pairs of N—H⋯N hydrogen bonds, forming inversion dimers, which are reinforced by pairs of C—H⋯S hydrogen bonds. The dimers are linked *via* C—H⋯π inter­actions, forming ribbons along [010].

## Related literature   

For the crystal structure of the 4-methyl derivative, 4-methyl-*N*-{[2-(pyridin-2-yl)eth­yl]carbamo­thio­yl}benzamide, see: Adam *et al.* (2014 ▸). For the crystal structure of *N*-carbamo­thioyl-2-methyl­benzamide, see: Adam *et al.* (2015 ▸).
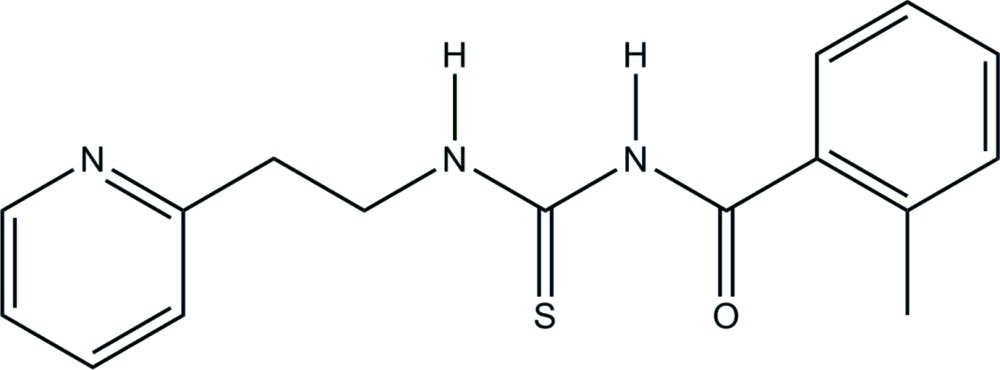


## Experimental   

### Crystal data  

C_16_H_17_N_3_OS
*M* *_r_* = 299.38
Triclinic, 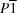

*a* = 8.5434 (4) Å
*b* = 8.7477 (4) Å
*c* = 11.0530 (5) Å
α = 86.1868 (13)°
β = 83.3739 (13)°
γ = 73.8746 (13)°
*V* = 787.73 (6) Å^3^
*Z* = 2
Mo *K*α radiation
μ = 0.21 mm^−1^
*T* = 100 K
0.53 × 0.42 × 0.22 mm

### Data collection  

Bruker APEX DUO CCD area-detector diffractometer
Absorption correction: multi-scan (*SADABS*; Bruker, 2009 ▸) *T*_min_ = 0.670, *T*_max_ = 0.867
30520 measured reflections
4698 independent reflections
3412 reflections with *I* > 2σ(*I*)
*R*_int_ = 0.045

### Refinement  

*R*[*F* ^2^ > 2σ(*F* ^2^)] = 0.045
*wR*(*F* ^2^) = 0.129
*S* = 1.03
4698 reflections
199 parameters
H atoms treated by a mixture of independent and constrained refinement
Δρ_max_ = 0.37 e Å^−3^
Δρ_min_ = −0.22 e Å^−3^

### 

Data collection: *APEX2* (Bruker, 2009 ▸); cell refinement: *SAINT* (Bruker, 2009 ▸); data reduction: *SAINT*; program(s) used to solve structure: *SHELXS2013* (Sheldrick, 2008 ▸); program(s) used to refine structure: *SHELXL2014* (Sheldrick, 2015 ▸); molecular graphics: *SHELXTL* (Sheldrick, 2008 ▸) and *Mercury* (Macrae *et al.*, 2008 ▸); software used to prepare material for publication: *SHELXL2014* and *PLATON* (Spek, 2009 ▸).

## Supplementary Material

Crystal structure: contains datablock(s) I, New_Global_Publ_Block. DOI: 10.1107/S2056989015013559/su5173sup1.cif

Structure factors: contains datablock(s) I. DOI: 10.1107/S2056989015013559/su5173Isup2.hkl

**Fig. 1.**
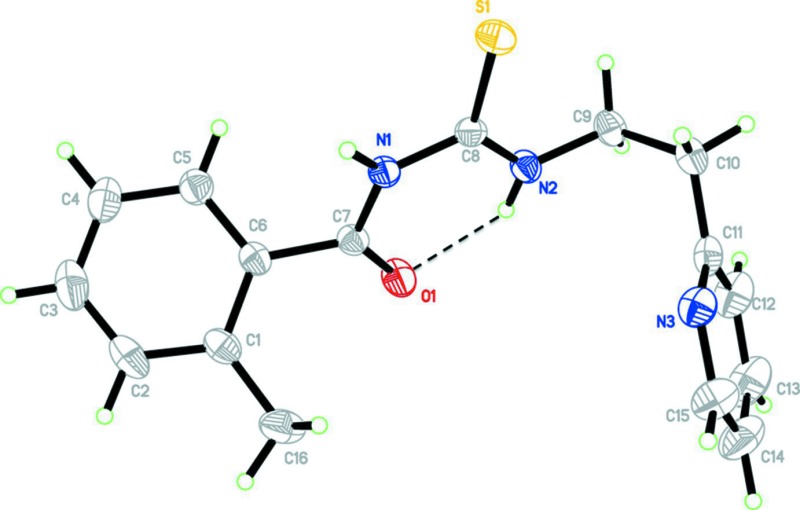
The molecular structure of the title compound, with atom labelling. Displacement ellipsoids are drawn at the 50% probability level. The intramolecular N-H···O hydrogen bond is shown as a dashed line (see Table 1 for details).

**Fig. 2.**
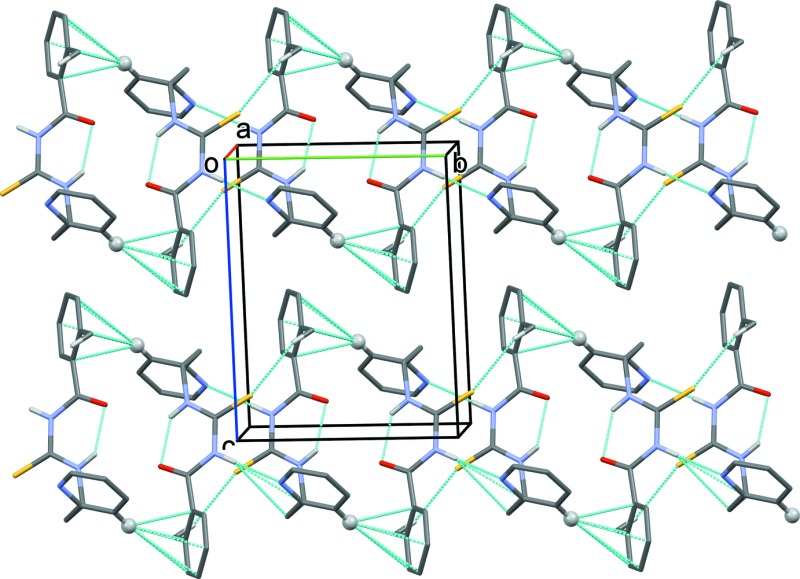
A view along the a axis of the crystal packing of the title compound. The hydrogen bonds and C-H···π interactions (H atom a grey ball) are shown as dashed lines (see Table 1 for details).

. DOI: 10.1107/S2056989015013559/su5173fig1.tif
The mol­ecular structure of the title compound, with atom labelling. Displacement ellipsoids are drawn at the 50% probability level. The intra­molecular N-H⋯O hydrogen bond is shown as a dashed line (see Table 1 for details).

a . DOI: 10.1107/S2056989015013559/su5173fig2.tif
A view along the *a* axis of the crystal packing of the title compound. The hydrogen bonds and C-H⋯π inter­actions (H atom a grey ball) are shown as dashed lines (see Table 1 for details).

CCDC reference: 1412857

Additional supporting information:  crystallographic information; 3D view; checkCIF report

## Figures and Tables

**Table 1:** Hydrogen-bond geometry (, ) *Cg*2 is the centroid of the C1C6 ring.

*D*H*A*	*D*H	H*A*	*D* *A*	*D*H*A*
N2H1*N*2O1	0.842(17)	1.971(18)	2.6550(16)	137.8(16)
N1H1*N*1N3^i^	0.875(17)	2.061(17)	2.9366(17)	180(3)
C16H16*A*S1^i^	0.96	2.86	3.782(2)	162
C12H12*A* *Cg*2^ii^	0.93	2.92	3.7724(19)	153

Symmetry codes: (i) 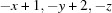; (ii) 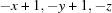.

## supplementary crystallographic information

### S1. Synthesis and crystallization

Ortho Benzoyl chloride (13 mmol) was added drop wise to a stirred acetone solution (30 ml) of ammonium thio­cyanate (13 mmol). Stirring was continued for 10 min. A solution of ethyl pyridine in acetone was then added and the reaction mixture was refluxed for 3 h, after which the solution was poured into a beaker containing some ice cubes. The resulting precipitate was collected by titration, washed several times with a cold ethanol/water mixture and purified by recrystallization from an ethanol solution, yielding colourless plate-like crystals.

### S1.1. Refinement

Crystal data, data collection and structure refinement details are summarized in Table 2. The NH H-atoms were located in a difference Fourier map and freely refined. The C-bound H atoms were positioned geometrically and refined using a riding model: C—H = 0.93–0.96 Å with U_iso_(H) = 1.5U_eq_(C) for methyl H atoms and 1.2U_eq_(C) for other H atoms.

### S2. Structural commentary

\ The title compound, Fig. 1, is a benzoyl thio­urea derivative with the pyridine and benzene rings being inclined to one another by 66.54 (9) °. The bond lengths and angles are very similar to those observed for the analogous structure, 4-methyl-N-[2-(pyridin-2-yl)ethyl­carbamo­thio­yl]\ benzamide (Adam *et al.*, 2014). However, in the 4-methyl derivative the pyridine and benzene rings are inclined to one another by 71.33 (15) °. There is an intra­molecular N—H···O hydrogen bond present in both molecules forming an S(6) ring motif (for the title compound, see Table 1 and Fig. 1).

In the crystal, molecules are linked *via* pairs of N—H···N hydrogen bonds forming inversion dimers which are reinforced by pairs of C—H···S hydrogen bonds (Fig. 2 and Table 1). The dimers are linked *via* C—H···π inter­actions forming ribbons along [010]; Table 1 and Fig. 2.

### Crystal data

**Table:**

C_16_H_17_N_3_OS	*Z* = 2
*M**_r_* = 299.38	*F*(000) = 316
Triclinic, *P*1	*D*_x_ = 1.262 Mg m^−^^3^
*a* = 8.5434 (4) Å	Mo *K*α radiation, λ = 0.71073 Å
*b* = 8.7477 (4) Å	Cell parameters from 8347 reflections
*c* = 11.0530 (5) Å	θ = 2.4–28.3°
α = 86.1868 (13)°	µ = 0.21 mm^−^^1^
β = 83.3739 (13)°	*T* = 100 K
γ = 73.8746 (13)°	Plate, colourless
*V* = 787.73 (6) Å^3^	0.53 × 0.42 × 0.22 mm

### Data collection

**Table:**

Bruker APEX DUO CCD area-detector diffractometer	4698 independent reflections
Radiation source: fine-focus sealed tube	3412 reflections with *I* > 2σ(*I*)
Graphite monochromator	*R*_int_ = 0.045
φ and ω scans	θ_max_ = 30.3°, θ_min_ = 1.9°
Absorption correction: multi-scan (*SADABS*; Bruker, 2009)	*h* = −12→12
*T*_min_ = 0.670, *T*_max_ = 0.867	*k* = −12→12
30520 measured reflections	*l* = −15→15

### Refinement

**Table:**

Refinement on *F*^2^	Primary atom site location: structure-invariant direct methods
Least-squares matrix: full	Secondary atom site location: difference Fourier map
*R*[*F*^2^ > 2σ(*F*^2^)] = 0.045	Hydrogen site location: mixed
*wR*(*F*^2^) = 0.129	H atoms treated by a mixture of independent and constrained refinement
*S* = 1.03	*w* = 1/[σ^2^(*F*_o_^2^) + (0.0515*P*)^2^ + 0.2342*P*] where *P* = (*F*_o_^2^ + 2*F*_c_^2^)/3
4698 reflections	(Δ/σ)_max_ < 0.001
199 parameters	Δρ_max_ = 0.37 e Å^−^^3^
0 restraints	Δρ_min_ = −0.22 e Å^−^^3^

### Special details

**Table:**

Geometry. All e.s.d.'s (except the e.s.d. in the dihedral angle between two l.s. planes) are estimated using the full covariance matrix. The cell e.s.d.'s are taken into account individually in the estimation of e.s.d.'s in distances, angles and torsion angles; correlations between e.s.d.'s in cell parameters are only used when they are defined by crystal symmetry. An approximate (isotropic) treatment of cell e.s.d.'s is used for estimating e.s.d.'s involving l.s. planes.

### Fractional atomic coordinates and isotropic or equivalent isotropic displacement parameters (Å^2^)

**Table:**

	*x*	*y*	*z*	*U*_iso_*/*U*_eq_	
S1	0.29858 (5)	1.05179 (5)	−0.14484 (4)	0.05473 (14)	
O1	0.46415 (15)	0.62159 (12)	0.10945 (10)	0.0552 (3)	
N1	0.32417 (14)	0.87622 (13)	0.05803 (10)	0.0367 (2)	
N2	0.47718 (15)	0.75403 (15)	−0.11337 (11)	0.0408 (3)	
N3	0.87338 (16)	0.81820 (15)	−0.15598 (13)	0.0499 (3)	
C1	0.3515 (2)	0.72438 (18)	0.36636 (14)	0.0482 (3)	
C2	0.2522 (3)	0.7379 (2)	0.47681 (15)	0.0654 (5)	
H2A	0.3012	0.7083	0.5488	0.079*	
C3	0.0847 (3)	0.7934 (3)	0.48282 (18)	0.0731 (6)	
H3A	0.0226	0.7999	0.5581	0.088*	
C4	0.0088 (2)	0.8393 (2)	0.37890 (18)	0.0638 (5)	
H4A	−0.1046	0.8769	0.3828	0.077*	
C5	0.10295 (19)	0.82906 (18)	0.26820 (15)	0.0478 (3)	
H5A	0.0522	0.8619	0.1972	0.057*	
C6	0.27224 (18)	0.77065 (15)	0.26075 (12)	0.0392 (3)	
C7	0.36489 (17)	0.74777 (16)	0.13728 (12)	0.0384 (3)	
C8	0.37507 (16)	0.88350 (16)	−0.06595 (12)	0.0359 (3)	
C9	0.5275 (2)	0.73791 (19)	−0.24280 (13)	0.0462 (3)	
H9A	0.4348	0.7906	−0.2875	0.055*	
H9B	0.5589	0.6259	−0.2610	0.055*	
C10	0.6698 (2)	0.80764 (19)	−0.28709 (14)	0.0487 (3)	
H10A	0.6926	0.7974	−0.3746	0.058*	
H10B	0.6371	0.9203	−0.2709	0.058*	
C11	0.82339 (18)	0.73009 (17)	−0.22889 (14)	0.0445 (3)	
C12	0.9112 (2)	0.5738 (2)	−0.2508 (2)	0.0661 (5)	
H12A	0.8745	0.5138	−0.3019	0.079*	
C13	1.0517 (3)	0.5084 (2)	−0.1970 (3)	0.0805 (7)	
H13A	1.1105	0.4032	−0.2102	0.097*	
C14	1.1056 (2)	0.5992 (3)	−0.1232 (2)	0.0779 (6)	
H14A	1.2021	0.5582	−0.0867	0.093*	
C15	1.0123 (2)	0.7523 (2)	−0.1053 (2)	0.0676 (5)	
H15A	1.0477	0.8142	−0.0549	0.081*	
C16	0.5335 (2)	0.6686 (3)	0.36477 (18)	0.0661 (5)	
H16A	0.5808	0.7494	0.3264	0.099*	
H16B	0.5743	0.5728	0.3200	0.099*	
H16C	0.5624	0.6477	0.4468	0.099*	
H1N2	0.508 (2)	0.676 (2)	−0.0647 (16)	0.049 (5)*	
H1N1	0.265 (2)	0.967 (2)	0.0876 (16)	0.049 (5)*	

### Atomic displacement parameters (Å^2^)

**Table:**

	*U*^11^	*U*^22^	*U*^33^	*U*^12^	*U*^13^	*U*^23^
S1	0.0565 (2)	0.0474 (2)	0.0519 (2)	−0.00366 (17)	−0.00764 (18)	0.01618 (17)
O1	0.0692 (7)	0.0369 (5)	0.0446 (6)	0.0061 (5)	0.0018 (5)	0.0038 (4)
N1	0.0395 (6)	0.0309 (5)	0.0360 (6)	−0.0043 (4)	−0.0032 (4)	0.0010 (4)
N2	0.0463 (6)	0.0384 (6)	0.0355 (6)	−0.0096 (5)	−0.0016 (5)	0.0026 (5)
N3	0.0483 (7)	0.0395 (6)	0.0586 (8)	−0.0079 (5)	−0.0005 (6)	−0.0052 (6)
C1	0.0641 (9)	0.0415 (7)	0.0405 (7)	−0.0166 (7)	−0.0103 (7)	0.0054 (6)
C2	0.0994 (15)	0.0627 (11)	0.0355 (8)	−0.0253 (10)	−0.0080 (9)	0.0049 (7)
C3	0.0886 (15)	0.0695 (12)	0.0523 (10)	−0.0179 (11)	0.0201 (10)	−0.0027 (9)
C4	0.0601 (10)	0.0592 (10)	0.0635 (11)	−0.0100 (8)	0.0140 (9)	−0.0032 (8)
C5	0.0506 (8)	0.0436 (8)	0.0458 (8)	−0.0086 (6)	−0.0015 (6)	−0.0008 (6)
C6	0.0499 (8)	0.0310 (6)	0.0357 (7)	−0.0099 (5)	−0.0038 (6)	0.0006 (5)
C7	0.0430 (7)	0.0331 (6)	0.0368 (7)	−0.0067 (5)	−0.0054 (5)	0.0006 (5)
C8	0.0345 (6)	0.0375 (6)	0.0369 (6)	−0.0111 (5)	−0.0072 (5)	0.0028 (5)
C9	0.0559 (9)	0.0488 (8)	0.0360 (7)	−0.0176 (7)	−0.0025 (6)	−0.0056 (6)
C10	0.0598 (9)	0.0483 (8)	0.0374 (7)	−0.0171 (7)	0.0017 (6)	0.0012 (6)
C11	0.0474 (8)	0.0380 (7)	0.0449 (8)	−0.0131 (6)	0.0112 (6)	−0.0009 (6)
C12	0.0633 (11)	0.0428 (9)	0.0883 (14)	−0.0116 (8)	0.0089 (10)	−0.0158 (9)
C13	0.0581 (11)	0.0424 (9)	0.128 (2)	0.0000 (8)	0.0105 (12)	−0.0077 (11)
C14	0.0437 (9)	0.0661 (12)	0.1145 (18)	−0.0032 (9)	−0.0051 (10)	0.0084 (12)
C15	0.0534 (10)	0.0612 (11)	0.0871 (14)	−0.0109 (8)	−0.0126 (9)	−0.0062 (10)
C16	0.0702 (12)	0.0708 (12)	0.0607 (11)	−0.0217 (9)	−0.0261 (9)	0.0174 (9)

### Geometric parameters (Å, º)

**Table:**

S1—C8	1.6675 (13)	C5—H5A	0.9300
O1—C7	1.2236 (16)	C6—C7	1.4926 (19)
N1—C7	1.3686 (17)	C9—C10	1.525 (2)
N1—C8	1.3919 (17)	C9—H9A	0.9700
N1—H1N1	0.874 (18)	C9—H9B	0.9700
N2—C8	1.3203 (18)	C10—C11	1.496 (2)
N2—C9	1.4499 (18)	C10—H10A	0.9700
N2—H1N2	0.841 (19)	C10—H10B	0.9700
N3—C11	1.331 (2)	C11—C12	1.387 (2)
N3—C15	1.336 (2)	C12—C13	1.364 (3)
C1—C2	1.396 (2)	C12—H12A	0.9300
C1—C6	1.397 (2)	C13—C14	1.372 (3)
C1—C16	1.494 (3)	C13—H13A	0.9300
C2—C3	1.372 (3)	C14—C15	1.369 (3)
C2—H2A	0.9300	C14—H14A	0.9300
C3—C4	1.366 (3)	C15—H15A	0.9300
C3—H3A	0.9300	C16—H16A	0.9600
C4—C5	1.378 (2)	C16—H16B	0.9600
C4—H4A	0.9300	C16—H16C	0.9600
C5—C6	1.388 (2)		
			
C7—N1—C8	127.33 (12)	N2—C9—H9A	108.9
C7—N1—H1N1	118.0 (12)	C10—C9—H9A	108.9
C8—N1—H1N1	114.5 (12)	N2—C9—H9B	108.9
C8—N2—C9	123.54 (13)	C10—C9—H9B	108.9
C8—N2—H1N2	116.3 (12)	H9A—C9—H9B	107.7
C9—N2—H1N2	120.0 (12)	C11—C10—C9	113.91 (13)
C11—N3—C15	118.03 (15)	C11—C10—H10A	108.8
C2—C1—C6	116.77 (16)	C9—C10—H10A	108.8
C2—C1—C16	120.27 (16)	C11—C10—H10B	108.8
C6—C1—C16	122.91 (15)	C9—C10—H10B	108.8
C3—C2—C1	122.25 (17)	H10A—C10—H10B	107.7
C3—C2—H2A	118.9	N3—C11—C12	121.26 (16)
C1—C2—H2A	118.9	N3—C11—C10	117.10 (13)
C4—C3—C2	120.40 (17)	C12—C11—C10	121.64 (16)
C4—C3—H3A	119.8	C13—C12—C11	119.68 (19)
C2—C3—H3A	119.8	C13—C12—H12A	120.2
C3—C4—C5	118.99 (18)	C11—C12—H12A	120.2
C3—C4—H4A	120.5	C12—C13—C14	119.45 (18)
C5—C4—H4A	120.5	C12—C13—H13A	120.3
C4—C5—C6	121.16 (16)	C14—C13—H13A	120.3
C4—C5—H5A	119.4	C15—C14—C13	117.6 (2)
C6—C5—H5A	119.4	C15—C14—H14A	121.2
C5—C6—C1	120.41 (14)	C13—C14—H14A	121.2
C5—C6—C7	118.19 (13)	N3—C15—C14	123.9 (2)
C1—C6—C7	121.25 (13)	N3—C15—H15A	118.0
O1—C7—N1	123.59 (13)	C14—C15—H15A	118.0
O1—C7—C6	121.89 (12)	C1—C16—H16A	109.5
N1—C7—C6	114.47 (11)	C1—C16—H16B	109.5
N2—C8—N1	117.23 (12)	H16A—C16—H16B	109.5
N2—C8—S1	124.66 (11)	C1—C16—H16C	109.5
N1—C8—S1	118.08 (10)	H16A—C16—H16C	109.5
N2—C9—C10	113.56 (12)	H16B—C16—H16C	109.5
			
C6—C1—C2—C3	0.1 (3)	C9—N2—C8—N1	173.37 (12)
C16—C1—C2—C3	177.89 (19)	C9—N2—C8—S1	−4.5 (2)
C1—C2—C3—C4	−0.6 (3)	C7—N1—C8—N2	−1.7 (2)
C2—C3—C4—C5	0.0 (3)	C7—N1—C8—S1	176.26 (11)
C3—C4—C5—C6	1.1 (3)	C8—N2—C9—C10	84.03 (18)
C4—C5—C6—C1	−1.6 (2)	N2—C9—C10—C11	61.39 (18)
C4—C5—C6—C7	174.02 (15)	C15—N3—C11—C12	0.8 (2)
C2—C1—C6—C5	0.9 (2)	C15—N3—C11—C10	−178.50 (15)
C16—C1—C6—C5	−176.76 (16)	C9—C10—C11—N3	−113.16 (15)
C2—C1—C6—C7	−174.53 (14)	C9—C10—C11—C12	67.54 (19)
C16—C1—C6—C7	7.8 (2)	N3—C11—C12—C13	−0.1 (3)
C8—N1—C7—O1	5.5 (2)	C10—C11—C12—C13	179.20 (17)
C8—N1—C7—C6	−171.85 (12)	C11—C12—C13—C14	−1.0 (3)
C5—C6—C7—O1	−128.16 (16)	C12—C13—C14—C15	1.2 (3)
C1—C6—C7—O1	47.4 (2)	C11—N3—C15—C14	−0.5 (3)
C5—C6—C7—N1	49.22 (18)	C13—C14—C15—N3	−0.5 (4)
C1—C6—C7—N1	−135.22 (14)		

### Hydrogen-bond geometry (Å, º)

Cg2 is the centroid of the C1–C6 ring.

**Table:**

*D*—H···*A*	*D*—H	H···*A*	*D*···*A*	*D*—H···*A*
N2—H1*N*2···O1	0.842 (17)	1.971 (18)	2.6550 (16)	137.8 (16)
N1—H1*N*1···N3^i^	0.875 (17)	2.061 (17)	2.9366 (17)	180 (3)
C16—H16*A*···S1^i^	0.96	2.86	3.782 (2)	162
C12—H12*A*···*Cg*2^ii^	0.93	2.92	3.7724 (19)	153

Symmetry codes: (i) −*x*+1, −*y*+2, −*z*; (ii) −*x*+1, −*y*+1, −*z*.
