# Supplementary figures and images for: Crystal structure of 2-methyl-N-{[2-(pyri­din-2-yl)eth­yl]carbamo­thio­yl}benzamide
Source: Acta Crystallogr E Crystallogr Commun. 2015 Aug 6;71(Pt 9):o636. doi: 10.1107/S2056989015013559 (PMC4555406; doi:10.1107/S2056989015013559)

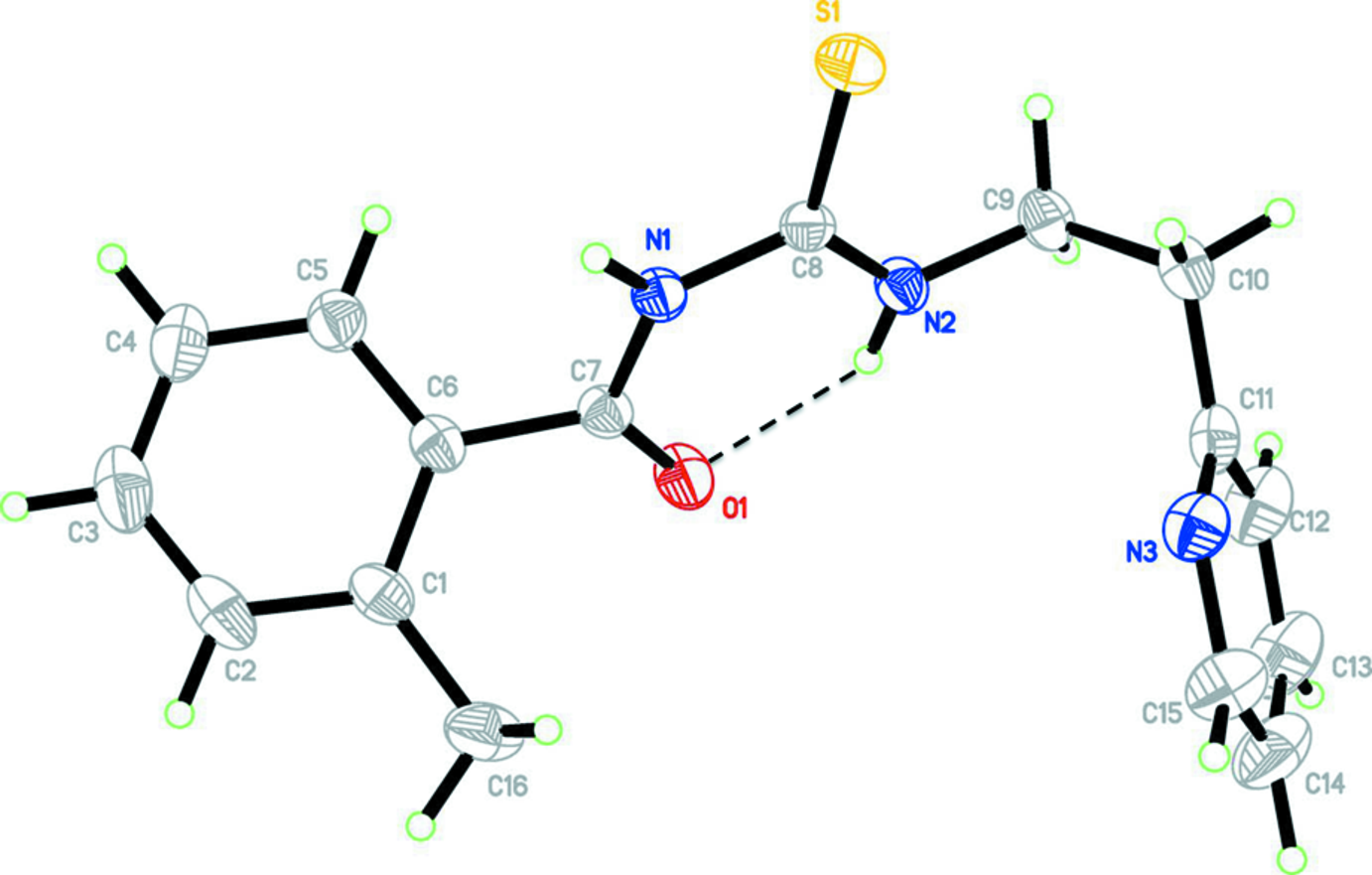

Supplement: Supplementary file 3 [file e-71-0o636-fig1.tif]

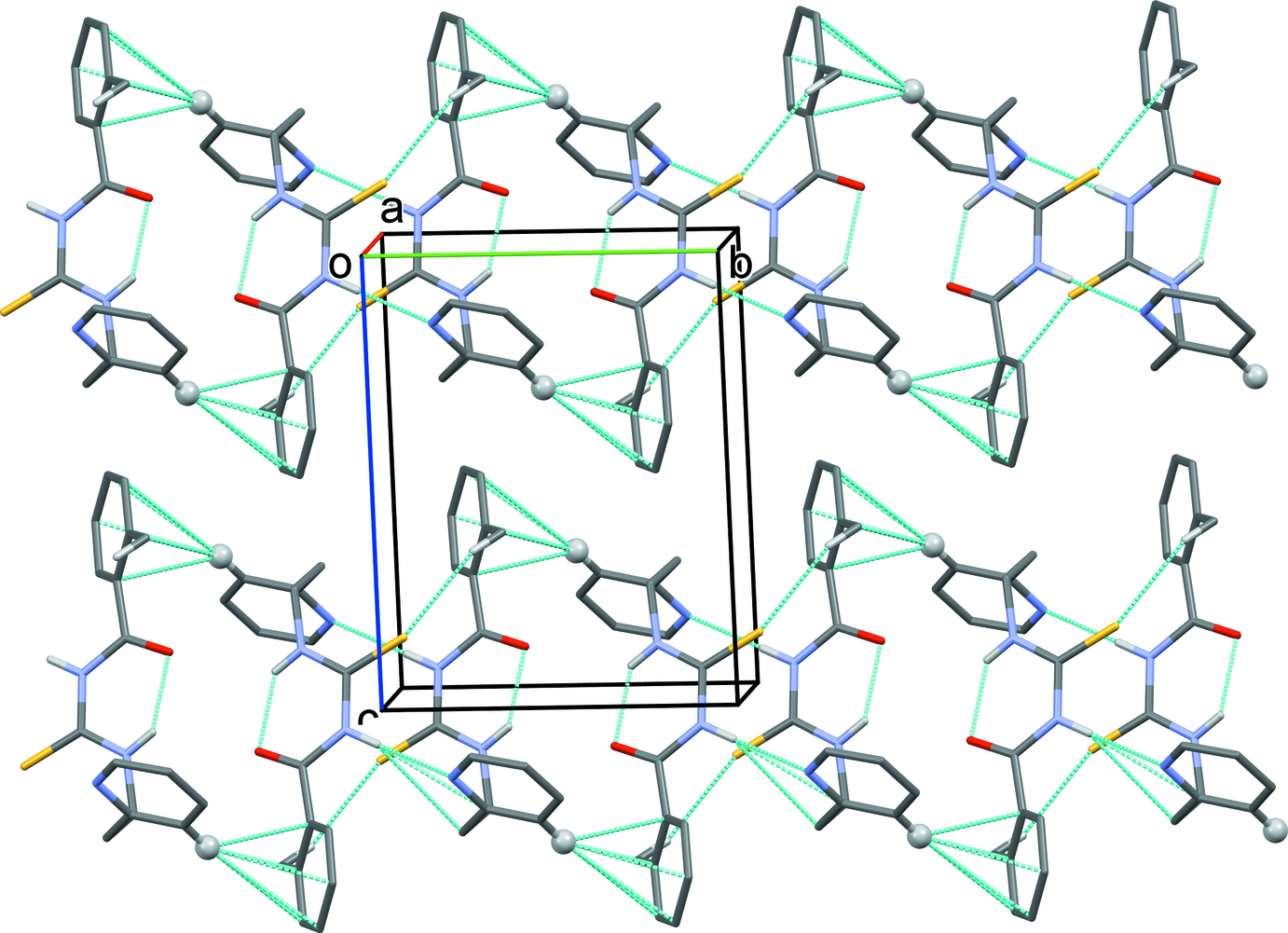

Supplement: Supplementary file 4 [file e-71-0o636-fig2.tif]
